# Prevalence of Grey Matter Pathology in Early Multiple Sclerosis Assessed by Magnetization Transfer Ratio Imaging

**DOI:** 10.1371/journal.pone.0024969

**Published:** 2011-09-15

**Authors:** Lydie Crespy, Wafaa Zaaraoui, Mathias Lemaire, Audrey Rico, Anthony Faivre, Françoise Reuter, Irina Malikova, Sylviane Confort-Gouny, Patrick J. Cozzone, Jean Pelletier, Jean-Philippe Ranjeva, Bertrand Audoin

**Affiliations:** 1 Pôle de Neurosciences Cliniques, Service de Neurologie, Assistance Publique Hôpitaux de Marseille, CHU Timone, Marseille, France; 2 Centre de Résonance Magnétique Biologique et Médicale (CRMBM) UMR CNRS 6612, Faculté de Médecine, Université de la Méditerranée, Aix-Marseille II, Marseille, France; University of Illinois at Chicago, United States of America

## Abstract

The aim of the study was to assess the prevalence, the distribution and the impact on disability of grey matter (GM) pathology in early multiple sclerosis. Eighty-eight patients with a clinically isolated syndrome with a high risk developing multiple sclerosis were included in the study. Forty-four healthy controls constituted the normative population. An optimized statistical mapping analysis was performed to compare each subject's GM Magnetization Transfer Ratio (MTR) imaging maps with those of the whole group of controls. The statistical threshold of significant GM MTR decrease was determined as the maximum p value (p<0.05 FDR) for which no significant cluster survived when comparing each control to the whole control population. Using this threshold, 51% of patients showed GM abnormalities compared to controls. Locally, 37% of patients presented abnormalities inside the limbic cortex, 34% in the temporal cortex, 32% in the deep grey matter, 30% in the cerebellum, 30% in the frontal cortex, 26% in the occipital cortex and 19% in the parietal cortex. Stepwise regression analysis evidenced significant association (p = 0.002) between EDSS and both GM pathology (p = 0.028) and T2 white matter lesions load (p = 0.019). In the present study, we evidenced that individual analysis of GM MTR map allowed demonstrating that GM pathology is highly heterogeneous across patients at the early stage of MS and partly underlies irreversible disability.

## Introduction

Pathological studies have clearly demonstrated that multiple sclerosis (MS) is an inflammatory disease of the central nervous system (CNS) characterized by macroscopic inflammatory lesions of white matter (WM) and grey matter (GM) associated with a diffuse microscopic inflammatory process of the CNS [1]. Extension of MS related pathology outside the well-known macroscopic lesions of the WM may explain the low relationship between disability progression and macroscopic WM lesions accrual [2]. In particular, the existence of GM pathology is now recognized as a pathological feature of MS [3]. In vivo, several MR studies have evidenced GM pathology in patients with different phenotypes of MS using several methods like magnetization transfer ratio (MTR) imaging [4], [5], [6], [7], [8], [9], [10], [11], diffusion weighted imaging [12], [13] or measures of atrophy [14]. Although the large majority of these studies have explored patients suffering from MS for several years, some recent studies using MTR or voxel based morphometry imaging have demonstrated that GM pathology occurs from the onset of the disease [4], [7], [14], [15]. Unfortunately, these techniques have not been optimized to give sensitive individual assessments of GM pathology according to their inability to clearly separate pathological from normal values at the individual level.

Development of method enable to quantify the extent of GM pathology at the individual level is now required for a potential application to the clinical practice. Recently, double inversion recovery (DIR) technique has provided for the first time in MS an individual map of the intra-cortical lesions (ICLs) [16]. DIR appears sensitive to evidence focal inflammatory ICLs in more than half of the MS population and in about one third of the patients at the first stage of the disease [17]. However, the ability of DIR to visualize the overall GM pathology is still suboptimal. Indeed, compared to histopathological data, DIR imaging is poorly sensitive to detect all the intracortical lesions [16], [18]. In addition, this technique does not explore the GM pathology located outside the ICLs and does not provide any quantification of the GM destructuration [17]. In contrast, MTR imaging provides a quantitative assessment of GM pathology located inside the macroscopic as well as the microscopic lesions of the GM.

In the present study, we performed a statistical analysis at the individual level of MTR maps to assess the prevalence, the distribution and the impact on disability of GM pathology in early multiple sclerosis.

## Materials and Methods

### Subjects

Eighty-eight patients presenting with a CIS and 44 healthy control subjects were included in this study. All the participants gave their written informed consent to participating in this study, which was approved by the local Ethics Committee (CPP Sud Méditerrané I). Patients were recruited at the Department of Neurology (University Hospital of Marseille), based on the following criteria: 1) age between 16 and 45; 2) occurrence of the first presumed inflammatory demyelinating event in the central nervous system (CSF) during the last twelve months, involving either the optic nerve, the spinal cord, the brainstem or a brain hemisphere; 3) no previous history of neurological symptom; 4) no possible alternative diagnosis; 5) a minimum delay since the corticosteroid infusion of two months; 6) ≥1 lesion in each of ≥2 characteristic locations of the CNS (periventricular, juxtacortical, posterior fossa, spinal cord) or 1 lesion in one characteristic location combined with oligoclonal bands in the CSF. The last criteria meant that only CIS patients with a high risk developing MS were included.

Patients underwent a clinical examination during the first neurological episode. Another neurological examination was performed on the day of MRI performed at least two months after the clinical episode. All the patients' disability levels were rated using the expanded disability status scale (EDSS) by the same neurologist on the day of MRI [19].

### MRI exploration

Patients and controls were imaged with a 1.5 Tesla commercially available unit (Magnetom Vision Plus; Siemens, Erlangen, Germany). The MR imaging protocol included localizer scout imaging, transverse fast spin-echo proton density-weighted and T_2_- weighted sequences (2600/15/85 ms [TR/TE1/TE2], 44 contiguous sections, 3-mm section thickness, 90° flip angle, 240-mm FOV, 256×256 matrix), and transverse proton density-weighted spoiled gradient-echo sequences (500/4.7 ms [TR/TE], 44 contiguous sections, 3-mm section thickness, 30° flip angle, 240-mm FOV, 256×256 matrix) performed without and with magnetization transfer (MT) saturation (1.5-kHz off-water resonance, 500°).

### Image processing

Segmentation processing could be disrupted by the presence of WM injury (focal demyelinating lesions). To prevent this pitfall, T_2_ lesions were first delineated onto the T2-weighted images by means of MRIcro program to obtain a T2-lesion mask. Mmt images were coregistered on T2-weighted images and the mean value of the WM signal outside lesions on Mmt images was transferred in areas where the T2 lesions were delineated. Then, “corrected” Mmt images without lesions were created to provide GM and WM masks not disrupted by the presence of WM injury. MTR maps were calculated on a voxel-by-voxel basis according to the following equation: MTR (%)  =  ((M_0_ - M_mt_)/M_0_)*100 where M_0_ and M_mt_ were the images obtained without and with MT saturation pulse, respectively. Then, we performed segmentation of MTR maps using the GM and WM masks obtained previously. Finally, GM MTR maps obtained were normalised into the MNI space using the T_2_ anatomical template provided by the SPM5 program and smoothed using a 12 mm Gaussian filter.

We used a similar images processing method for healthy controls except for the steps associated to the lesions.

### Statistical mapping analysis

The statistical threshold was defined as the maximum p value enabling to show no significant cluster when comparing any individual control with the whole population of controls (two-sample t-test, SPM5). This conservative criterion allows minimizing the false positive cluster potentially observed in patients.

Then, we performed the comparison between GM MTR maps of each patient and GM MTR maps of the 44 controls using the statistical threshold previously obtained. We obtained individual statistical maps of differences observed between each patient and control group. Then, we determined for each patient the extent of total GM injury defined as the ratio between the volume of GM injury and the volume of total GM. We also quantified the extent of local GM injury for the different brain sub-regions. The sub-regions were delineated on the basis of a computerized atlas (Pick-atlas, SPM5) and included the frontal cortex, the parietal cortex, the temporal cortex, the occipital cortex, the limbic cortex, the deep GM and the cerebellum.

Stepwise regression model was used to evaluate the potential impact of T_2_ lesion load and GM MTR on disability (EDSS).

### Sensitivity of individual MTR statistical mapping analysis

To assess the sensitivity of the method, we built artificial abnormal MTR brain maps using data from 6 randomly selected healthy controls from which MTR percent units were decreased uniformly by 1 to 15%. This was done by substracting 0.01 to 0.15 from GM voxels of each GM MTR map (imcalc function of SPM). Statistical mapping analyses of these modified MTR maps were then assessed and quantified using the predefined threshold. Relative volumes of GM abnormalities according to the percentage of global MTR degradation were plotted for the six subjects to determine the sensitivity of the method.

### Assessment of the variability of regional GM MTR values in controls

In order to evaluate the local GM MTR variance in the control population, we computed a variance map of GM MTR of the whole control population. Regional mean values of the variance have been reported for each of the same brain sub-regions used to quantify regional WM and GM lesions.

## Results

### Clinical and conventional MRI findings

Patients' demographic, clinical characteristics are reported in Table 1. In the CIS population, fifty four out of the eighty eight patients (61%) fulfilled the McDonald 2010 criteria for MS [20].

**Table 1:** Demographic and clinical characteristics of patients.

	Gender (F/M)	Age (years) (mean, range)	EDSS (median,mean, range)	Time to the clinical onset(month) (mean, range)	T2 Lesion load (cm^3^) (mean, standard deviation)	Location of symptom			
						ON	SC	B	H
Patients n = 88	70/18	29 (16–45)	0 – 0.6 (0–2)	4.9 (2–12)	4.6 8	21	34	24	9

ON: Optic Nerve, SC: Spinal Cord, B: Brainstem, H: Hemisphere.

In patients, the mean total T2 lesions volume was 4.6 cm^3^ (SD = 8), the mean number of T2 lesions was 15.7 (SD = 13) and the mean number of gadolinium enhancing lesions was 2.3 (SD = 4.3).

Mean age of the controls was 25.8 years (range: 18 to 50 years).

### Statistical threshold

Individual analyses contrasting each control with the whole group of controls were performed. The maximum p value enabling to show no significant cluster when comparing any individual control with the whole population of controls was p<0.05 (FDR corrected; k = 10). Using this threshold the minimum T-value obtained for a significant cluster in a patient was 2.9.

### Sensitivity of individual MTR statistical mapping analysis

**Figure 1:**
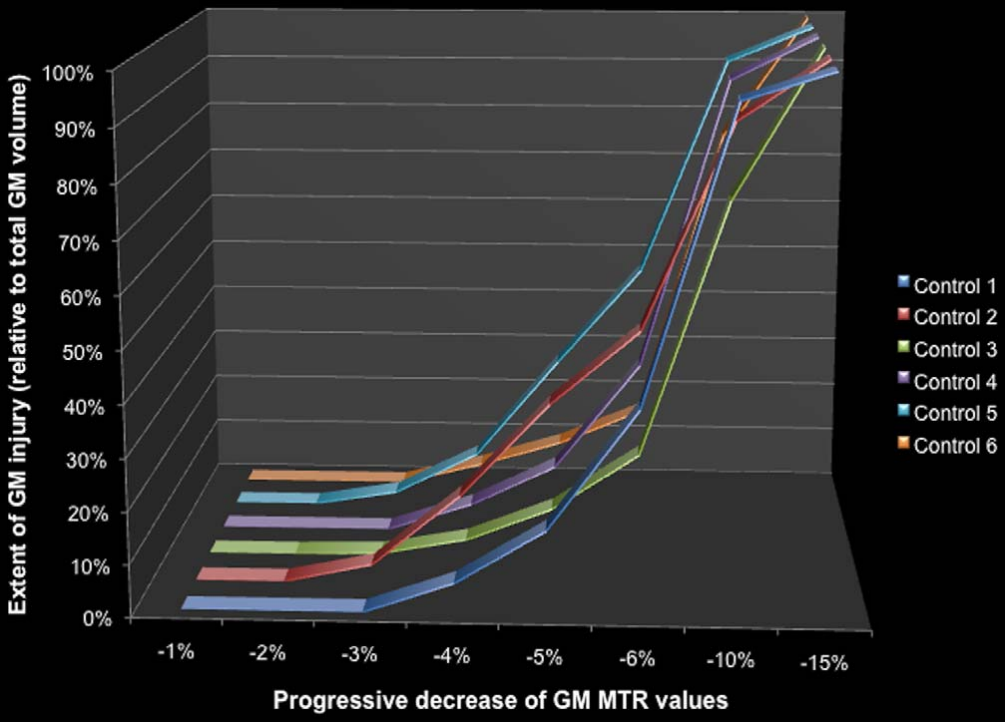
Sensitivity of individual MTR statistical mapping analysis. MTR maps of 6 controls were decreased uniformly in MTR by 1 to 15%. Statistical mapping analyses of these modified MTR maps were then assessed and quantified using the predefined threshold showing presence of clusters for all subjects for a 4% decrease in MTR.

At the same threshold used to detect local GM abnormalities in patients, (p<0.05, FDR corrected; k = 10), we observed significant clusters in the modified MTR maps of controls starting at 3% of artificial decrease in MTR for 3 out of the 6 controls. At 4% of MTR decrease all controls showed GM abnormalities (see Figure 1). This approach allowed demonstrating that patients showing GM MTR abnormalities at the selected threshold (p<0.05, FDR corrected; k = 10) showed a minimum regional MTR decreased of 3%.

### Variability of regional GM MTR value in controls

The mean values of the variance of GM MTR in controls was 2.4% (SD = 1.7%) in the frontal cortex, 3.2% (SD = 1.5%) in the parietal cortex, 2 % (SD = 1.7%) in the limbic cortex, 2.9% (SD = 1.6%) in the occipital cortex, 2.5% (1.6%) in the temporal cortex, 1.5 % (1.7%) in the deep GM and 1.2% (SD = 1.4%) in the cerebellum.

### Patterns of individual GM MTR decrease in patients

**Figure 2:**
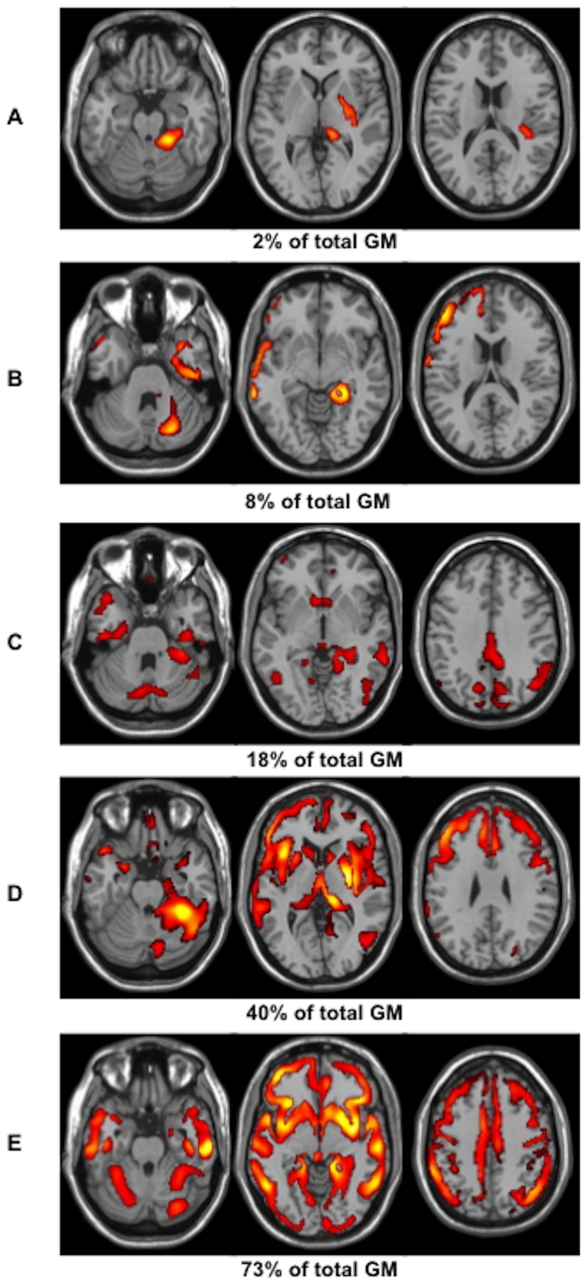
Statistical mapping of MTR abnormalities in five CIS patients (A–E) showing various extent of GM injury (range 2–73%).

Using the predefined threshold (p<0.05, FDR corrected; k = 10), forty-five out of the eighty-eight patients (51%) showed significant regional GM MTR decrease compared to controls. Examples of individual GM MTR maps obtained in five patients have been displayed in Figure 2.

**Figure 3:**
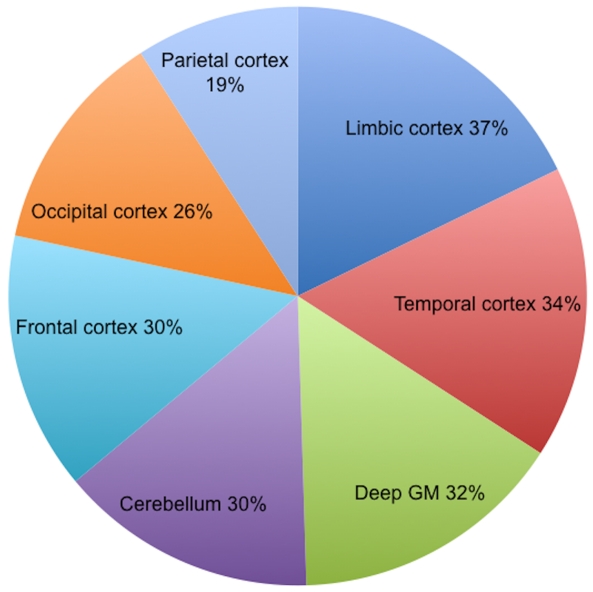
Regional distribution of GM injury among the whole group of CIS patients (n = 88). Values represent the proportion of patients showing GM injury within each sub-region.

**Figure 4:**
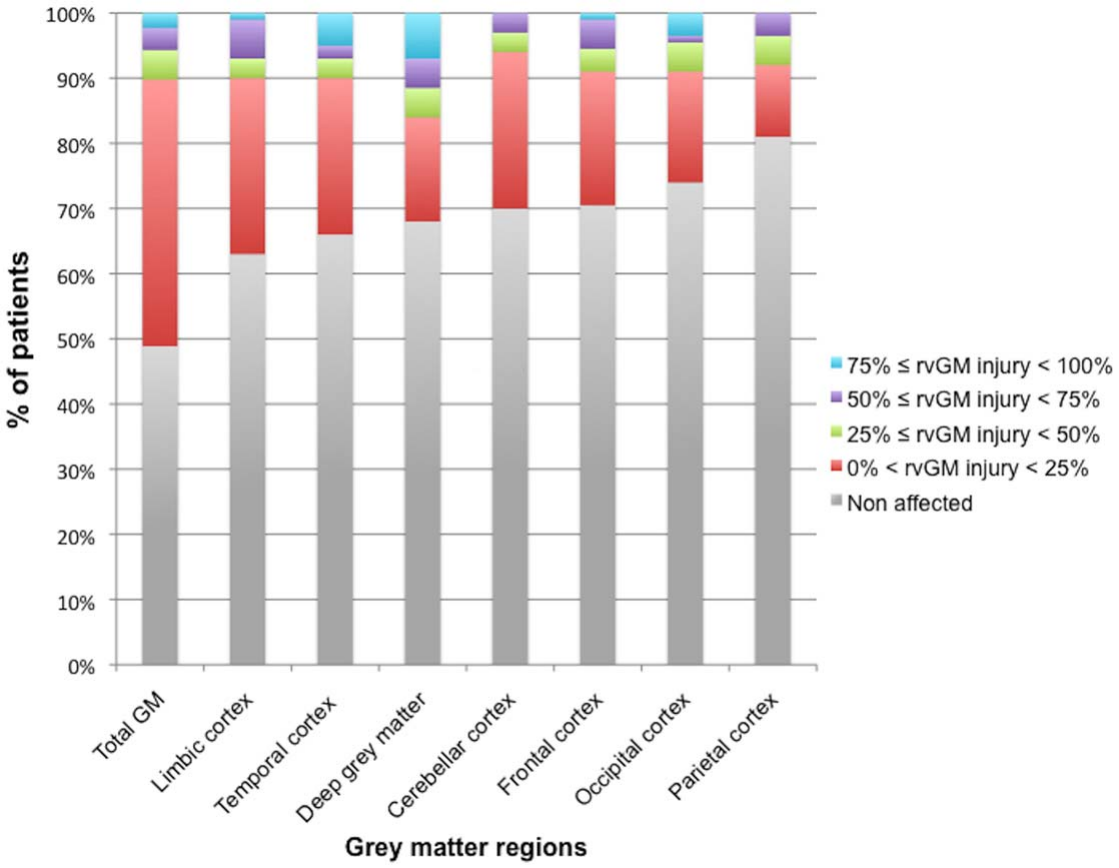
Proportion of affected GM regions in each brain regions in CIS patients. (rvGM injury: relative volume of GM injury).

Among all patients, 37% presented abnormalities inside the limbic cortex, 34% in the temporal cortex, 32% in the deep GM, 30% in the cerebellum, 30% in the frontal cortex, 26% in the occipital cortex and 19% in the parietal cortex (Figure 3). The distribution of patients according to the relative volume of affected GM within global and regional GM tissue (limbic cortex, temporal cortex, deep GM, cerebellum, frontal cortex, occipital cortex and parietal cortex) has been assessed (Figure 4). In the majority of the patients showing significant regional GM MTR decrease, the relative extent of regional GM injury not exceeded 25% of the volume of the respective region (Figure 4).

### Correlation between the extent of GM MTR decrease and conventional MRI findings in patients

The extent of GM MTR abnormalities was significantly correlated with the global T2 lesion load (Spearman rank correlation, p = 0.0006) and with the total number of T2 lesions (Spearman rank correlation, p = 0.0047). There is a trend between the number of gadolinium enhancing lesions and the extent of GM MTR abnormalities (Spearman rank correlation, p = 0.06).

T2 lesion load in the limbic region was correlated with the extent of GM MTR abnormalities in the limbic cortex (Spearman rank correlation, p = 0.02). T2 lesions load in the deep white matter was correlated with the extent of GM MTR abnormalities in the deep GM (Spearman rank correlation, p = 0.02). T2 lesions load in the parietal region was correlated with the extent of GM MTR abnormalities in the parietal cortex (Spearman rank correlation, p = 0.02). T2 lesions load in the frontal region was not correlated with the extent of GM MTR abnormalities in the frontal cortex (Spearman rank correlation, p = 0.18). T2 lesions load in the occipital region was not correlated with the extent of GM MTR abnormalities in the occipital cortex (Spearman rank correlation, p = 0.07). T2 lesions load in the cerebellum was not correlated with the extent of GM MTR abnormalities in the cerebellar cortex (Spearman rank correlation, p = 0.33).

### Correlation between the extent of GM MTR decrease, conventional MRI findings and clinical characteristics in patients

Stepwise regression analysis evidenced significant association (p = 0.002, R^2^ = 0.135) of EDSS with both grey matter pathology (p = 0.028) and T2 white matter lesions load (p = 0.019).

The extent of regional GM MTR decrease was not influenced by the type of the first clinical symptom (optic neuritic, myelitis, brainstem or hemispheric symptoms) : cerebellum (ANOVA, p = 0.33), deep GM (ANOVA, p = 0.83), frontal cortex (ANOVA, p = 0.75), limbic cortex (ANOVA, p = 0.65), occipital cortex (ANOVA, p = 0.47), parietal cortex (ANOVA, p = 0.25) and the temporal cortex (ANOVA, p = 0.65).

## Discussion

In the present study, an individual quantification of GM pathology has been applied in a large group of patients at the onset of MS and provides for the first time the prevalence, the distribution and the impact on disability of GM pathology in early multiple sclerosis.

### Assessment of GM pathology at the individual level

Recently, quantitative MRI methods have highlighted the existence of GM pathology in patients suffering from MS [21]. However, these methods applied at the group level, have not been designed to quantify the extent of GM pathology at the individual level preventing any potential application for the clinical practice. In contrast, individual GM MTR mapping quantifies the GM pathology for each MS subject [22] evidencing potential subtle GM MTR decrease (minimum 3%) at the individual level.

Using this method, we demonstrated the existence of GM injury in about half of patients at the earliest stage of the disease. Using DIR imaging, Calabrese et al evidenced the existence of GM lesions one third of CIS patients [17]. The apparent lower sensitivity of the DIR technique to provide the overall GM pathology may be related to its lower sensitivity to detect GM injury outside the macroscopic GM lesions [17]. In contrast, MTR provides a more global reflect of GM pathology demonstrating abnormalities inside the macroscopic as well as the microscopic lesions of the GM while it can not separate these two components. Therefore, combination of these two techniques, DIR and individual MTR GM maps analysis, may better reflect the real extent of GM pathology occurring in MS.

### Prevalence and pattern of GM injury at the onset of MS

The present study clearly evidenced that GM injury is common from the onset of MS and may involved all the GM regions of the brain. Few previous studies have assessed the distribution of GM pathology at this stage of the disease [8], [14], [23], [24]. They have used group analysis and only reflected the collocation of GM injury in the population of patients but not the distribution of GM pathology for each subject. They evidenced that the more frequent mutual locations of GM injury were the deep GM structures and the limbic system. In contrast to these group analyses, the present method, using an individual approach, provides the common pattern of GM injury but also its variability across patients. Using this method, we demonstrated that GM pathology is highly heterogeneous across patients. Indeed, only half of patients show GM abnormalities and among them, the volume of tissue injury is highly variable. In addition, we evidenced that GM pathology may occur in all brain regions with a lesser frequency in the parietal and occipital cortices.

Different mechanisms may contribute to GM pathology underlying MTR decrease. First, the existence of ICLs has been clearly demonstrated by histopathological and MRI studies [3], [25]. Secondly, axonal impairments resulting from WM lesions may induce distal GM lesions secondary to Wallerian degeneration or anterograde transynaptic damage in some regions particularly connected [26], [27], [28], [29]. The correlation found between the extent of GM MTR abnormalities and the T2 lesion load supports this hypothesis. Finally, a neurodegenerative process characterized by iron deposition may contribute to GM pathology particularly in the deep GM [30], [31]. However, a post-mortem MRI study using high field MRI performed in MS patients has evidenced that MTR value in GM lesions was significantly associated with the degree of demyelination and not with neuronal density. MTR imaging may be more sensitive to the inflammatory demyelinating lesions of the cortex compared to the neuronal pathology secondary to distant WM injury [32].

### Relationship between individual extent of GM injury and disability

MTR imaging has already been used to demonstrate the existence of correlations between GM injury and irreversible disability in CIS patients [4], [6], [7], [8], [9], [10], [33], [34]. These previous studies used extracted MTR values from GM masks and tested correlations with clinical parameters among the global populations of patients. These MTR values were affected both by physiological and pathological processes. In contrast, depiction of local GM pathology at the individual level using a validated voxel based statistical threshold determined in a control population, provides, similarly to the depiction of GM lesions using DIR, a quantitative parameter reflecting individual GM pathology. We suggest that this parameter could be a relevant surrogate marker of disease burden.

The impact of early GM pathology on irreversible disability may partly explain the clinico-radiological paradox observed with conventional MRI [2]. Indeed, as demonstrated in the present study, both WM lesions and GM injury contribute to disability. Although significant, the correlations found between tissue injury and EDSS remain modest as evidenced by Calabrese et al [17]. Several factors could explain the weakness of this association. Firstly, CIS patients showed either no or limited disability consistent with their short disease duration (the mean time from the first clinical episode was 5 months). Secondly, EDSS scale is particularly sensitive to spinal cord pathology that is often not explored. Finally, compensatory processes known to occur from the very first stage of the disease onwards may often mask the clinical impact of early GM pathology [35].

### Conclusion

In the present study, we evidenced that individual analysis of GM MTR map allowed demonstrating that GM pathology is highly heterogeneous across patients at the early stage of MS and partly underlies irreversible disability.
